# Clustering-based identification of immune-related gene signatures in hepatocellular carcinoma

**DOI:** 10.3332/ecancer.2025.2017

**Published:** 2025-10-17

**Authors:** Jyoti Brahmaiah, Usha Adiga, Alfred J Augustine, Sampara Vasishta

**Affiliations:** 1 Department of Pathology, Apollo Institute of Medical Sciences and Research Chittoor, Murukambattu, Chittoor 517127, Andhra Pradesh, India; 2 Department of Biochemistry, Apollo Institute of Medical Sciences and Research Chittoor, Murukambattu, Chittoor 517127, Andhra Pradesh, India; 3 Department of Surgery, Apollo Institute of Medical Sciences and Research Chittoor, Murukambattu, Chittoor 517127, Andhra Pradesh, India

**Keywords:** hepatocellular carcinoma, immune clustering, MHC genes, antigen presentation, immunotherapy

## Abstract

**Background:**

Hepatocellular carcinoma (HCC) is a complex malignancy influenced by genetic, epigenetic and immune-related factors. The tumour immune microenvironment plays a critical role in HCC progression and response to immunotherapy. Identifying key immune-related gene signatures through clustering techniques can provide insights into tumour biology and therapeutic targets.

**Methods:**

We employed K-means, Markov Clustering Algorithm (MCL) and density-based spatial clustering of applications with noise (DBSCAN) to analyse immune-related genes in HCC. Functional enrichment analysis was conducted using Gene Ontology (GO) biological process, cellular component and molecular function categories, along with pathway analysis from Kyoto encyclopedia of genes and genomes (KEGG) and Reactome databases. Additionally, protein–protein interaction (PPI) hub analysis and microRNAs (miRNA) target predictions were integrated to understand the regulatory networks.

**Results:**

K-means clustering segregated immune genes into three clusters, with major histocompatibility complex (MHC) class II genes forming a distinct cluster. MCL and DBSCAN identified a more unified immune cluster incorporating both MHC class I and II molecules, suggesting their coordinated role in antigen presentation. GO analysis revealed enrichment in antigen processing and presentation pathways, immunoglobulin-mediated responses and glutamate receptor signaling. KEGG pathway analysis highlighted associations with autoimmune diseases and viral infections. PPI hub analysis identified CD4, CD74 and HLA-DQA1 as central nodes, while miRNA analysis suggested regulatory interactions affecting immune gene expression.

**Conclusion:**

Our clustering analysis highlights distinct immune-related gene signatures in HCC, emphasising the role of antigen presentation and immune modulation in tumour progression. The findings provide a foundation for further investigation into immunotherapeutic strategies targeting key immune pathways in HCC.

## Introduction

Hepatocellular carcinoma (HCC) is the most prevalent form of primary liver cancer and represents a significant global health burden. It is strongly associated with chronic liver diseases, including hepatitis B virus (HBV) and hepatitis C virus (HCV) infections, Metabolic dysfunction-associated steatotic liver disease (MASLD) and alcohol-related liver disease [1] Although the present incidence (2.15 per 100,000), prevalence (2.27 per 100,000) and mortality (2.21 per 100,000) rates of HCC in India are lower than the global figures, the yearly rates of change in these measures are greater in India [2].

### Epidemiology and risk factors

The incidence of HCC varies globally, with higher rates observed in regions with endemic HBV and HCV infections, such as East Asia and sub-Saharan Africa [3]. Chronic HBV infection remains the leading cause of HCC, contributing to approximately 50% of cases worldwide, while chronic HCV infection accounts for 25%–30% of cases [4]. In recent years, MASLD has emerged as a critical risk factor, particularly in Western countries, where obesity and metabolic syndrome are prevalent [5]. Other risk factors include excessive alcohol consumption, aflatoxin exposure, diabetes and genetic predisposition [6].

### Molecular pathogenesis of HCC

HCC arises through a multistep process involving genetic and epigenetic alterations that drive malignant transformation. Key molecular pathways implicated in HCC development include Wnt/β-catenin signaling, TP53 mutations, oxidative stress and inflammation-mediated hepatocarcinogenesis [7]. The integration of HBV DNA into the host genome is a well-established oncogenic driver that promotes genomic instability and tumour progression [8]. Additionally, mutations in genes such as TERT, CTNNB1 and TP53 are frequently observed in HCC tumours, further highlighting the heterogeneous nature of the disease [9].

### Role of genetic variants in HCC development

Genome-wide association studies have identified several genetic variants associated with increased susceptibility to HCC. The PNPLA3 rs738409 C>G polymorphism has been strongly linked to MASLD-associated HCC, with studies demonstrating that carriers of the G allele exhibit increased hepatic fat accumulation and fibrosis progression [10]. Similarly, TM6SF2 rs58542926 has been associated with hepatic steatosis and an elevated risk of fibrosis, contributing to HCC development in patients with MASLD [11]. Other significant variants, such as MBOAT7 rs641738 and HSD17B13 rs72613567, have been implicated in modifying liver disease progression and influencing HCC susceptibility [12,13].

### Objectives

1. To analyse the biological processes (BPs), cellular components (CCs) and molecular functions (MFs) of clustered immune genes, focusing on antigen presentation, major histocompatibility complex (MHC) class I and II interactions and immune response pathways in HCC.
2. To explore key immune pathways enriched in HCC, including PD-1 signaling, antigen processing and presentation, interferon gamma signaling and glutamate receptor signaling, to assess their role in immune evasion and tumour progression.
3. To investigate the involvement of microRNAs (miRNAs) in HCC immune regulation, identify specific miRNAs that target immunerelated genes and contribute to immune modulation in the tumour microenvironment.
4. To identify protein–protein interaction (PPI) hub proteins linked to immune regulation in HCC, highlighting key molecules that may serve as central regulators of immune response and potential therapeutic targets.
5. To compare the clustering effectiveness of K-means, Markov Clustering Algorithm (MCL) and density-based spatial clustering of applications with noise (DBSCAN) in classifying immune-related genes in HCC, and evaluate the similarities and differences between these approaches in capturing biologically relevant gene interactions.

## Methodology

### Study design and data acquisition

This study employed a bioinformatics-driven approach to analyse HCC data, focusing on gene expression, pathway enrichment and protein interaction networks. The dataset was obtained from publicly available genomic repositories [14], including gene expression Omnibus and the cancer genome atlas. The selection criteria included datasets with well-defined sample classifications, including HCC tumour tissues and adjacent normal liver tissues. The inclusion of high-throughput sequencing and microarray data allowed for a comprehensive assessment of gene expression variations, molecular interactions and pathway deregulations.

### Differential gene expression analysis

Differential expression analysis was conducted using DESeq2 for RNA-Seq data and limma for microarray data. Genes with an adjusted *p*-value (false discovery rate, FDR) of <0.05 and a log2 fold change of >1 or <−1 were considered significantly differentially expressed. The lists of upregulated and downregulated genes were further validated by cross-referencing with existing literature on HCC.

### Functional enrichment analysis

Gene ontology (GO) enrichment and Kyoto encyclopedia of genes and genomes (KEGG) pathway analysis were performed using ClusterProfiler in R. The GO analysis categorised the significantly expressed genes into three domains: BP, CC and MF. The KEGG analysis identified significantly enriched pathways related to oncogenesis, immune response and metabolic dysregulation in HCC.

For immune-related processes, GO terms such as ‘Antigen Processing and Presentation via MHC Class II’ were analysed to determine the role of HLA-DQA1 in tumour immune evasion. Additionally, pathways related to neurotransmitter signaling, such as ‘Glutamate Receptor Signaling’, were assessed for their involvement in HCC progression.

### PPI network construction

To identify key hub proteins and their potential role in hepatocarcinogenesis, a PPI network was constructed using STRING and visualised using Cytoscape. The MCODE algorithm was applied to detect highly interconnected clusters of proteins. Key hub proteins such as FLNA, replication timing regulatory factor 1 (RIF1) and DLG4 were identified based on their high degree of connectivity.

### miRNA target prediction and regulatory network analysis

To assess post-transcriptional regulation in HCC, miRNA enrichment analysis was conducted using miRTarBase 2017 and TargetScan databases. Significant interactions were identified between HLA-DQA1 and miRNAs such as hsa-miR-6798-3p and hsa-miR-4645-5p, which have been implicated in immune modulation. Similarly, hsa-miR-122-5p, a liver-specific miRNA, was examined for its role in tumour progression.

The regulatory network was constructed by integrating mRNA, miRNA and PPI interactions, providing insights into how miRNAs influence oncogenic pathways in HCC.

### Statistical analysis

All statistical analyses were performed using R (version 4.1.2) and Python (version 3.9). A *p*-value of <0.05 was considered statistically significant. Multiple testing correction was applied using the Benjamini–Hochberg (BH) method to control for false discovery rates.

In this study, we applied three clustering techniques—K-means, MCL and DBSCAN—to classify immune-related genes associated with HCC.

### Ethical considerations

This is not a clinical case report but a bioinformatics-based analysis using publicly available datasets from repositories. No direct patient recruitment, intervention or identifiable clinical data were involved; therefore, informed consent and institutional ethical approval were not required. All datasets used were generated under the ethical guidelines of their respective repositories.

## Results

### GO BP analysis

**Figure 1.**
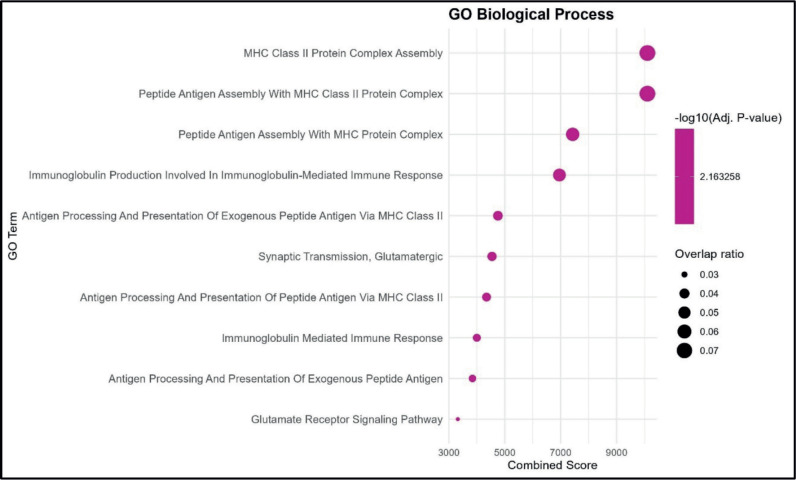
GO BP enrichment analysis. Bubble plot showing the top enriched GO BP terms. The *x*-axis represents the combined enrichment score, and the *y*-axis lists the GO terms. Bubble size corresponds to the overlap ratio (proportion of query genes overlapping with genes in each GO term), and bubble color intensity reflects the statistical significance as –log₁₀ (adjusted *p*-value).

GO enrichment analysis identified several BP significantly associated with the dataset. The highest combined scores were observed for MHC Class II protein complex assembly and peptide antigen assembly with MHC Class II protein complex, both showing strong enrichment and relatively high overlap ratios. Other notable processes included peptide antigen assembly with MHC protein complex and immunoglobulin production involved in the immunoglobulin-mediated immune response, indicating a clear link to antigen presentation and immune activation pathways. Processes related to antigen processing and presentation of exogenous peptide antigen via MHC Class II, synaptic transmission (glutamatergic) and glutamate receptor signaling pathway were also enriched, though with comparatively lower combined scores and overlap ratios. Overall, the enrichment profile highlights a predominance of immune-related pathways, particularly those linked to MHC Class II-mediated antigen processing and presentation. This suggests that the dataset is functionally enriched for processes central to adaptive immune responses (Figure 1).

### GO CC analysis

The enrichment analysis for CCs indicated the strongest associations with MHC Class II protein complex and MHC protein complex, both showing the highest combined scores and overlap ratios. Additional enriched components included the lumenal side of the endoplasmic reticulum membrane and the ionotropic glutamate receptor complex, suggesting a combination of immune-related and neuronal membrane-associated elements.

**Figure 2.**
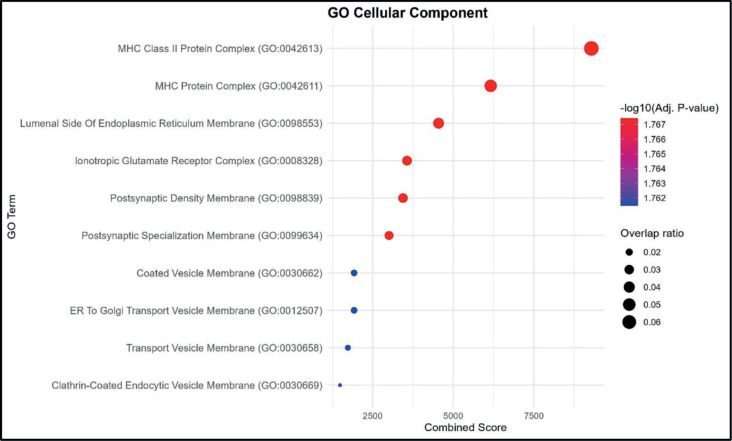
GO CC enrichment analysis. Bubble plot showing the top enriched GO BP terms. The *x*-axis represents the combined enrichment score, and the *y*-axis lists the GO terms. Bubble size corresponds to the overlap ratio (proportion of query genes overlapping with genes in each GO term), and bubble color intensity reflects the statistical significance as –log₁₀ (adjusted *p*-value).

Other terms with notable enrichment were postsynaptic density membrane and postsynaptic specialisation membrane, reflecting potential involvement of synaptic structures. Lower combined score terms such as coated vesicle membrane, ER to Golgi transport vesicle membrane, transport vesicle membrane and clathrin-coated endocytic vesicle membrane indicate processes linked to intracellular transport and vesicular trafficking. The dataset is enriched for membrane-associated complexes, with predominant representation of MHC-related immune structures and secondary enrichment in synaptic and vesicular transport components. (Figure 2).

### GO MF analysis

**Figure 3.**
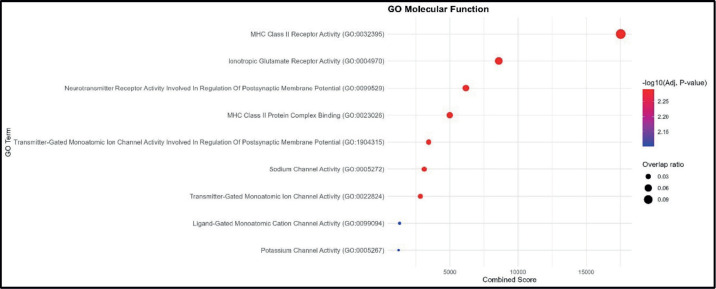
GO MF enrichment analysis. Bubble plot showing the top enriched GO BP terms. The *x*-axis represents the combined enrichment score, and the *y*-axis lists the GO terms. Bubble size corresponds to the overlap ratio (proportion of query genes overlapping with genes in each GO term), and bubble color intensity reflects the statistical significance as –log₁₀ (adjusted *p*-value).

The MF analysis identified MHC Class II receptor activity as the most enriched term, with the highest combined score and overlap ratio. This was followed by ionotropic glutamate receptor activity and neurotransmitter receptor activity involved in the regulation of postsynaptic membrane potential, indicating functional links to both immune recognition and neuronal signaling. Other enriched terms included MHC Class II protein complex binding and transmitter-gated monoatomic ion channel activity involved in the regulation of postsynaptic membrane potential, further highlighting the interplay between antigen presentation and synaptic signaling. Lower combined score functions, such as sodium channel activity, ligand-gated monoatomic cation channel activity and potassium channel activity, point toward additional ion transport processes. The dataset is enriched for functions related to MHC Class II-mediated antigen recognition and binding, with secondary enrichment in neurotransmitter receptor activity and ion channel regulation (Figure 3).

### KEGG pathway analysis

**Figure 4.**
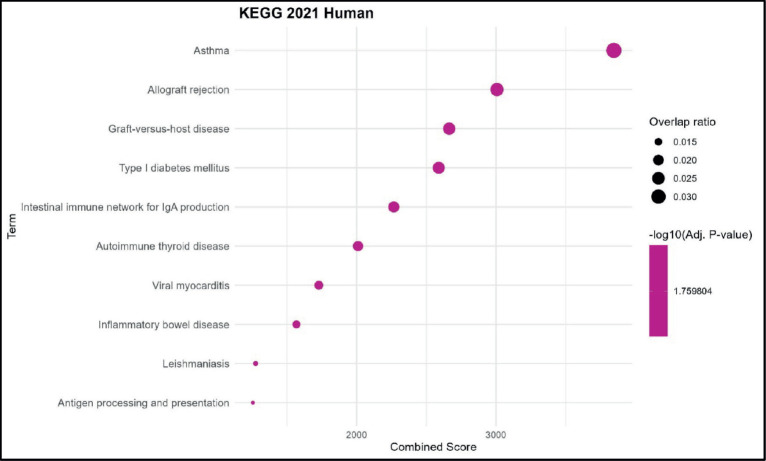
KEGG pathway enrichment analysis. Bubble plot showing the top enriched GO BP terms. The *x*-axis represents the combined enrichment score, and the *y*-axis lists the GO terms. Bubble size corresponds to the overlap ratio (proportion of query genes overlapping with genes in each GO term), and bubble color intensity reflects the statistical significance as –log₁₀ (adjusted *p*-value).

KEGG pathway analysis revealed significant enrichment in immune-related diseases, with the highest combined score observed for asthma, followed by allograft rejection and graft-versus-host disease. Other notable enriched pathways included type I diabetes mellitus, intestinal immune network for IgA production and autoimmune thyroid disease, highlighting strong associations with autoimmune and hypersensitivity conditions. Lower-ranked enriched terms comprised viral myocarditis, inflammatory bowel disease, leishmaniasis and antigen processing and presentation, indicating a broader involvement of the dataset in infectious and inflammatory immune pathways. The dataset shows predominant enrichment for KEGG pathways related to autoimmune, inflammatory and immune-mediated diseases, suggesting a central role in immune dysregulation and pathogen response (Figure 4).

### miRTarBase 2017 analysis

The enriched miRNAs are represented in Table 1.

### PPI hub proteins

PPI network analysis identified key hub proteins, with FLNA, RIF1 and DLG4 showing significant interactions with GRIK1 and HLA-DQA1. FLNA (Filamin A) plays a role in cytoskeletal organisation, while RIF1 is involved in DNA repair. These findings, represented in Table 2, suggest that alterations in these proteins could contribute to tumour progression and immune evasion in HCC. Tables 3–5 represent the various clustering methods, such as k-means, MCL and DBSCAN, respectively.

This bioinformatics analysis of HCC data provides a comprehensive understanding of the immune and neurological pathways implicated in disease progression. The enrichment of HLA-DQA1 in antigen processing pathways underscores its role in immune modulation, while GRIK1-associated neurotransmitter signaling highlights a potential novel mechanism in hepatocarcinogenesis. The identified miRNAs and hub proteins suggest additional regulatory layers influencing HCC pathophysiology. These findings provide valuable insights for future research, particularly in developing immunotherapy strategies and targeted treatments for HCC.

## Discussion

Our analysis of HCC using various bioinformatics tools provides critical insights into the immune-related molecular mechanisms contributing to disease progression. The GO terms, KEGG pathway analysis, PPI hub proteins and miRNA regulatory network collectively highlight key immune system components involved in HCC pathogenesis.

The KEGG pathway results further support the notion that autoimmune and inflammatory pathways are closely tied to HCC development. Additionally, miRNA regulation of key immune genes may serve as a novel mechanism for immune evasion, offering potential therapeutic targets. The clustering analysis revealed three major insights:

Class II Predominance in K-means: The K-means approach isolated MHC class II genes into a single cluster, indicating their distinct expression pattern in HCC. This highlights their role in adaptive immunity and tumour immune escape.

Integration of class I and II in MCL and DBSCAN: Unlike K-means, both MCL and DBSCAN clustered MHC class I and II genes together. This suggests functional cross-talk between these molecules in shaping the tumour immune microenvironment (TIME).

CD4 and CD74 as key regulators: The consistent clustering of CD4 and CD74 with MHC genes underscores their pivotal role in antigen presentation and T-cell activation in HCC.

Differential clustering of MHC class I and II genes, the clustering methods employed demonstrated distinct grouping tendencies of MHC genes. K-means clustering separated MHC class II molecules into a single group, while MCL and DBSCAN clustered both class I and II genes together. This distinction suggests that while class II molecules may exhibit distinct transcriptional or post-transcriptional regulation in HCC, their interaction with class I molecules remains essential in immune recognition and tumour evasion. The presence of class I molecules such as HLA-B within the same cluster as class II genes in MCL and DBSCAN points to functional convergence in antigen processing and immune activation.

CD4 and CD74 as central players in immune modulation, the consistent clustering of CD4 and CD74 with MHC class II genes underscores their critical role in antigen presentation and T-cell activation. CD4, a key co-receptor for MHC class II molecules, is central to T-helper cell function, supporting immune surveillance and cytokine-mediated responses. Similarly, CD74, which acts as an MHC class II chaperone, facilitates antigen processing and presentation. Their co-clustering with HLA genes reinforces their role in orchestrating the immune response against HCC cells, potentially influencing patient prognosis and treatment response.

Potential implications for tumour immune microenvironment, the clustering of immune-related genes reveals patterns that may reflect the tumour microenvironment's immunological landscape. The integration of MHC class I and II genes, as seen in MCL and DBSCAN clustering, indicates a coordinated immune response that may be influenced by tumour-induced immune suppression. The immune escape mechanisms in HCC often involve the downregulation of MHC molecules, allowing tumour cells to evade cytotoxic T-cell recognition. Understanding these clustering patterns could help identify novel biomarkers and therapeutic targets to counteract immune evasion strategies in HCC.

Comparative effectiveness of clustering techniques, the similarities between MCL and DBSCAN suggest these methods are robust for detecting biologically relevant interactions among immune genes. Both approaches grouped class I and II molecules together, capturing the functional interplay between these components. In contrast, K-means identified a distinct cluster for class II molecules, possibly due to differences in transcriptional regulation or specific cellular pathways unique to antigen-presenting cells. These variations highlight the importance of method selection when analysing immune-related gene expression in cancer research.

The TIME plays a crucial role in HCC progression and response to therapy. HCC tumours exhibit immune suppression through multiple mechanisms, including the upregulation of immune checkpoint molecules such as PD-1, CTLA-4 and TIM-3, which contribute to T-cell exhaustion [15]. Additionally, alterations in antigen presentation pathways, such as downregulation of MHC class I and II molecules, allow tumour cells to evade immune surveillance [16]. Understanding the immune landscape of HCC is essential for the development of immunotherapeutic strategies.

Recent advancements in computational biology have enabled the clustering of immune-related genes in HCC, shedding light on distinct immune subtypes with potential therapeutic implications. Clustering approaches such as K-means, MCL and DBSCAN have been utilised to classify immune-related genes based on their expression profiles [17]. These techniques have identified key immune clusters involving MHC class I and II molecules, CD4 and CD74, which are integral to antigen presentation and T-cell activation [18].

The identification of immune-related gene clusters has significant implications for personalised therapy in HCC. Immunotherapy, particularly immune checkpoint inhibitors (ICIs) targeting PD-1 and CTLA-4, has revolutionised HCC treatment [19]. The combination of ICIs, such as tremelimumab with durvalumab, has demonstrated promising efficacy in patients with unresectable HCC [20]. Additionally, therapies targeting the Wnt/β-catenin pathway and tumour-associated macrophages are being explored as potential strategies to enhance antitumour immunity [21].

## Limitations of the study

The findings presented in this study are derived entirely from *in silico* analyses, which, while powerful for identifying potential biological associations, are inherently hypothesis-generating in nature. As such, these results should be interpreted with caution, as they require experimental validation to confirm their biological relevance. Further studies using cellular and rodent models are essential to elucidate the underlying mechanisms and to assess causality. Moreover, validation in appropriate clinical cohorts will be necessary to determine the translational significance of these findings in human disease contexts.

## Conclusion

This study underscores the intricate interplay between the immune system and HCC, with a strong emphasis on antigen presentation, immune evasion and neuroimmune interactions. The identification of key regulatory miRNAs and hub proteins provides promising avenues for further research into targeted therapies. Future investigations should focus on validating these findings through experimental and clinical studies to improve our understanding of HCC pathogenesis and treatment strategies.

Our study demonstrates the utility of clustering algorithms in identifying immune gene interactions in HCC. The integration of MHC class I and II molecules in MCL and DBSCAN suggests coordinated immune regulation, while K-means highlights distinct expression patterns. These insights contribute to understanding immune modulation in HCC and may guide future immunotherapeutic strategies.

## Conflicts of interest

The authors declare no conflicts of interest related to this work.

## Funding

This research did not receive any specific grant from funding agencies in the public, commercial or not-for-profit sectors.

## Author contributions

Jyoti Brahmaiah – conceptualisation, data curation, formal analysis, original draft preparation; Usha Adiga – statistical analysis, methodology refinement, project administration, critical manuscript revision; Alfred J Augustine – supervision, validation, interpretation of results, critical feedback; Sampara Vasishta – literature review, bioinformatics analysis, visualisation, writing (review and editing), correspondence. All authors reviewed and approved the final manuscript.

## Declaration of generative AI and AI-assisted technologies in the writing process

During the preparation of the articles, the authors used AI tools to reformulate some sentences, after which the authors reviewed and edited the content as needed and take full responsibility of the content in the article.

## Figures and Tables

**Table 1.** Enrichment analysis of miRNAs. The table shows significant miRNAs identified through enrichment analysis. Columns represent the miRNA term, overlap of genes in the input list with the miRNA target set (input genes/miRNA target set size), raw *p*-value, adjusted *p*-value (BH correction), old *p*-value and old adjusted p-value from prior analysis, odds ratio (enrichment strength), combined score (integrating the *p*-value and odds ratio) and the specific overlapping gene(s).

Term	Overlap	*p*-value	Adjusted *p*-value	Old *p*-value	Old adjusted *p*-value	Odds ratio	Combined score	Genes
hsa-miR-6798-3p	1/28	0.0027980636576703	0.0111922546306812	0	0	739.6666666666666	4,348.372853162505	HLA-DQA1
hsa-miR-4645-5p	1/145	0.0144476715160197	0.0195282873353324	0	0	137.875	584.206985754933	HLA-DQA1
hsa-miR-4673	1/147	0.0146462155014993	0.0195282873353324	0	0	135.97260273972603	574.2902550659267	HLA-DQA1
hsa-miR-122-5p	1/610	0.0600709814635586	0.0600709814635586	0	0	31.83743842364532	89.5341482504527	HLA-DQA1

**Table 2.** Enrichment analysis of protein targets associated with identified genes. The table lists significantly enriched protein targets predicted to interact with the query genes. Columns indicate the protein target (Term), overlap of genes in the input set with the target’s associated genes (input genes/total target-associated genes), raw p-value, adjusted p-value (BH correction), old p-value and old adjusted p-value from previous analysis, odds ratio (measure of enrichment strength), combined score (integration of p-value and odds ratio) and the specific overlapping gene(s).

Term	Overlap	p-value	Adjusted p-value	Old p-value	Old adjusted p-value	Odds ratio	Combined score	Genes
FLNA	1/135	0.0134546517038701	0.0390965888145967	0	0	148.23880597014926	638.676575305457	GRIK1
RIF1	1/157	0.0156386355258387	0.0390965888145967	0	0	127.1923076923077	528.8669878232274	HLA-DQA1
DLG4	1/409	0.0404825893488036	0.0539530708707953	0	0	48.01470588235294	153.97755796464196	GRIK1
PRKACA	1/440	0.04351685936573	0.0539530708707953	0	0	44.55353075170843	139.65780243417447	GRIK1
PRKCA	1/547	0.0539530708707953	0.0539530708707953	0	0	35.62637362637363	104.01620930061436	GRIK1

**Table 3.** K-means clustering of proteins based on expression or interaction profiles. Proteins were grouped into clusters using the K-means method. Each entry lists the clustering method, assigned cluster number, number of genes in the cluster, protein name and full protein description. Cluster 1 contains nine proteins, predominantly HLA class II molecules, while clusters 2 and 3 each contain a single protein.

#Clustering method	Cluster number	Gene count	Protein name	Protein description
K-means	1	9	CD4	T-cell surface glycoprotein CD4
K-means	1	9	CD74	HLA class II histocompatibility antigen gamma chain
K-means	1	9	HLA-DMA	HLA class II histocompatibility antigen, DM alpha chain
K-means	1	9	HLA-DMB	HLA class II histocompatibility antigen, DM beta chain
K-means	1	9	HLA-DPA1	HLA class II histocompatibility antigen, DP alpha 1 chain
K-means	1	9	HLA-DPB1	HLA class II histocompatibility antigen, DP beta 1 chain
K-means	1	9	HLA-DQA1	MHC, class II, DQ alpha 1
K-means	1	9	HLA-DRA	HLA class II histocompatibility antigen, DR alpha chain
K-means	1	9	HLA-DRB1	HLA class II histocompatibility antigen, DRB1 beta chain
K-means	2	1	HLA-DOA	HLA class II histocompatibility antigen, DO alpha chain
K-means	3	1	HLA-B	HLA class I histocompatibility antigen, B alpha chain

**Table 4.** MCL analysis of proteins based on network connectivity. Proteins were grouped into clusters using the MCL method. Each entry lists the clustering method, cluster number, number of genes in the cluster, protein name and a brief functional description. Cluster 1 contains 11 proteins, with the majority representing HLA class II histocompatibility antigens, alongside CD4 and the HLA class I molecule HLA-B.

#Clustering method	Cluster number	Gene count	Protein name	Protein description
MCL	1	11	CD4	T-cell surface glycoprotein CD4
MCL	1	11	CD74	HLA class II histocompatibility antigen gamma chain
MCL	1	11	HLA-B	HLA class I histocompatibility antigen, B alpha chain
MCL	1	11	HLA-DMA	HLA class II histocompatibility antigen, DM alpha chain
MCL	1	11	HLA-DMB	HLA class II histocompatibility antigen, DM beta chain
MCL	1	11	HLA-DOA	HLA class II histocompatibility antigen, DO alpha chain
MCL	1	11	HLA-DPA1	HLA class II histocompatibility antigen, DP alpha 1 chain
MCL	1	11	HLA-DPB1	HLA class II histocompatibility antigen, DP beta 1 chain
MCL	1	11	HLA-DQA1	MHC, class II, DQ alpha 1; Belongs to the MHC class II family.
MCL	1	11	HLA-DRA	HLA class II histocompatibility antigen, DR alpha chain
MCL	1	11	HLA-DRB1	HLA class II histocompatibility antigen, DRB1 beta chain

**Table 5.** DBSCAN analysis of proteins. Proteins were grouped using the DBSCAN clustering method based on network proximity and density criteria. The table lists the clustering method, cluster number, gene count per cluster, protein name and functional description. Cluster 1 comprises 11 proteins, predominantly HLA class II histocompatibility antigens, alongside CD4 and the HLA class I molecule HLA-B.

#Clustering method	Cluster number	Gene count	Protein name	Protein description
DBSCAN	1	11	CD4	T-cell surface glycoprotein CD4
DBSCAN	1	11	CD74	HLA class II histocompatibility antigen gamma chain
DBSCAN	1	11	HLA-B	HLA class I histocompatibility antigen, B alpha chain
DBSCAN	1	11	HLA-DMA	HLA class II histocompatibility antigen, DM alpha chain
DBSCAN	1	11	HLA-DMB	HLA class II histocompatibility antigen, DM beta chain
DBSCAN	1	11	HLA-DOA	HLA class II histocompatibility antigen, DO alpha chain
DBSCAN	1	11	HLA-DPA1	HLA class II histocompatibility antigen, DP alpha 1 chain
DBSCAN	1	11	HLA-DPB1	HLA class II histocompatibility antigen, DP beta 1 chain
DBSCAN	1	11	HLA-DQA1	MHC, class II, DQ alpha 1
DBSCAN	1	11	HLA-DRA	HLA class II histocompatibility antigen, DR alpha chain
DBSCAN	1	11	HLA-DRB1	HLA class II histocompatibility antigen, DRB1 beta chain
